# Socioeconomic barriers to facility-based delivery in urban poor communities of Lagos: Wealth, linguistic capacity, and residential area

**DOI:** 10.1016/j.hpopen.2020.100019

**Published:** 2020-11-11

**Authors:** Yoshito Kawakatsu, Hirotsugu Aiga, Osy Ubani, Adefunke Oyeniyi Adesina, Sumihisa Honda, Junko Otaki, Nobuhiro Kadoi

**Affiliations:** a Department of Global Health, University of Washington, USA; b School of Tropical Medicine and Global Health, Nagasaki University, Nagasaki, Japan; c Human Development Department, Japan International Cooperation Agency, Tokyo, Japan; d Lagos State Ministry of Health, Lagos, Nigeria; e Graduate School of Biomedical Sciences, Nagasaki University, Nagasaki, Japan

**Keywords:** Antenatal care, Skilled birth attendance, Wealth, Language Linguistic barriers, Urban slum, Nigeria

## Abstract

**Background:**

The objective of this study is to describe delivery care utilizations and to identify socioeconomic barriers to practicing facility-based deliveries, in urban poor communities of Lagos, Nigeria.

**Methods:**

The study was a prospective cohort study conducted in Lagos Mainland, a local government area of Lagos State, Nigeria. A total of 723 randomly selected pregnant women participated in this study. The socio-economic barriers were sought from three different perspectives, i.e., household wealth as a financial barrier, linguistic capacity as a social barrier, and residential area as a physical barrier. The data were analyzed with descriptive and multivariate statistical methods using the imputed datasets.

**Results:**

While 41.9% of the participants delivered at governmental health facilities, 28.3% and 29.8% of them gave births at private health facilities and other places, respectively. As the results of the analyses, household wealth was positively associated with childbirth at a governmental health facility. Egun-speaking women were more likely to give births at private facilities than Yoruba-speaking women, while Igbo-speaking and Hausa-speaking women were more likely to choose other places for their childbirths.

**Conclusion:**

This study identified the financial and linguistic barriers to facility-based deliveries among women in urban poor communities.

## Highlights

• A representative of pregnant women in urban poor areas prospectively monitored.
• Antenatal care places were the significant predictors of delivery places.
• The wealthier women were more likely to deliver at governmental health facilities.
• Igbo- or Hausa-speaking women tended to utilize non-facility places for childbirth.
• The residential areas of pregnant women were not associated with delivery places.

## 1 Background

Reduction in maternal and child mortalities is one of the key priorities under the Sustainable Development Goal 3, *Ensure healthy lives and promote well-being for all at all ages,* being derived from earlier Millennium Development Goal (MDG) 4, MDG 5, and MDG 6. For over the past 25 years, maternal mortality ratio (MMR) was reduced by nearly 44% globally, through a series of intensive and continued interventions. Despite the efforts made, there remain a total of 303 thousand maternal deaths worldwide in 2015 [1]. Note that two-thirds of global maternal deaths in 2015 occurred in Sub-Sarahan Africa [1]. Nigeria is one of the countries having the greatest number of maternal deaths as of 2015.

The World Health Organization (WHO) recommends providing skilled professional midwifery services during delivery as a critical intervention for further reduction in maternal mortalities, stillbirths, and newborn mortalities [2]. Yet, in Nigeria, only 36% and 38% of deliveries were facility-based and assisted by skilled birth attendants (SBAs), respectively [3]. The gap in SBA attendance coverage between wealth quintiles and between mothers’ education levels were reported in the country [3], [4].

Financial barriers and their proxies (e.g., maternal employment and women’s decision-making autonomy) are frequently reported as the determinants of SBA attendance during delivery from Sub-Saharan African countries [5], [6], [7], [8], [9]. Other studies reported geographic access to health facilities as another key determinant of SBA attendance in those countries [10], [11], [12], [13]. There are few studies on the determinants of SBA attendance targeting the urban poor in Nigeria. The only related study we found attempted to identify the determinants of SBA attendance among those having visited clinics for Bacille Calmette-Guérin (BCG) immunization in Lagos [14]. Moreover, earlier studies having reported the linguistic barriers to health service utilizations (incl. SBA attendance) were limited to those having been conducted in high-income countries [15], [16], [17].

Thus, the determinants of SBA attendance among the urban poor in Nigeria remain unknown, despite the need for identifying them in view of growing urban poor populations in the country. This study is aimed at describing the realities of maternal service utilizations for childbirths in Lagos, Nigeria, the most populated city of all the seven megacities in Africa. It further attempts to identify socioeconomic barriers to practicing facility-based deliveries in urban poor communities of Lagos, from three perspectives, i.e., household wealth as a financial barrier, linguistic capacity as a social barrier, and residential area as a physical barrier. Thus, this study helps deepen the knowledge of pregnant women’s health-seeking behaviors and thereby develop evidence-based strategic options for increasing maternal health service utilizations.

## 2 Methods

### 2.1 Study site

Lagos State is located in the south-western part of Nigeria. It is the most populated city of all the seven megacities in Africa. Having been estimated at approximately 12 million as of 2014, the population of Lagos is projected to be doubled (i.e., 24 million) by 2030 [18]. Rapid urbanization due to both natural and social growths make it challenging for its subpopulations, particularly those living in urban slums, to access and utilize health services. As a result, both mortality and morbidity rates were becoming higher, particularly in urban poor communities of Lagos [19]. Discrepancies in health service coverages between local government areas (LGAs) were previously reported by the Lagos State Ministry of Health [20]. The report describes inequity in health care utilizations and thereby health status between the richer and the poor. The difference in SBA coverage between them was the greatest of all the major maternal and child health service coverages such as antenatal care and vaccination coverage [20].

Despite the increasing ethnic diversity among the populations in Lagos State [21], the majority of health workers at governmental health facilities in Lagos continue to be Yoruba, a major ethnic group of southern Nigeria. Although non-Yoruba populations are likely to have a certain level of challenges in communicating with health workers, there has been no earlier study focusing on the linguistic barriers to health service utilizations in Lagos State.

This study was conducted in Lagos Mainland LGA, one of 20 LGAs in Lagos State. According to the 2006 National Population and Housing Census Report [22], Lagos Mainland LGA accounts for 3.6% of the total population of Lagos State (326,700 people). The population of Lagos State as of 2016 was estimated at 449,900 in a total area of 19.5 square kilometers. The population density (23,071 people per km^2^) is one of the highest in Nigeria [23]. Its land area is located slightly above sea level. Thus, Lagos State is prone to flooding in the rainy season.

This study site was one of the target sites of the Japan International Cooperation Agency (JICA) technical cooperation project “Strengthening Pro-Poor Community Health Services in Lagos State.” The project started in Eti-Osa LGA of Lagos in 2014 and later expanded its implementation to Lagos Mainland LGA in 2017.

### 2.2 Study design and sampling procedure

This study was designed as a prospective cohort study in Lagos Mainland LGA (Appendix 1). The baseline and follow-up surveys were conducted in February 2017 and July 2018, respectively. Prior to the baseline survey, all the 861 enumeration areas (EAs) in Lagos Mainland LGA were visited by the surveyors and their assistants. Of the 861 EAs, 14 were in non-residential areas, 44 were primarily occupied by school buildings, and seven were demolished. In addition, it was not permitted to enter nine EAs located in gated estates for the purpose of the surveys. Therefore, 787 EAs were visited to identify pregnant women. As a result, a total of 2499 pregnant women were identified and listed. Of the 2499 pregnant women listed, 387 were not interviewed during the baseline survey (i.e., 54 refused, 81 moved, 48 unreachable, one deceased, and 203 having given births before the interview). Thus, the total number of participants recruited for the baseline survey was 2112.

At the follow-up survey, 1000 participants were randomly selected from the 2112 pregnant women having participated in the baseline survey. Of 1000, 734 mothers having undergone live births were interviewed. Seventy-eight mothers rejected, and 104 mothers were either relocated or unreachable. Two pregnant women died and 82 experienced miscarriage. Of the 734 mothers having undergone live birth, 36 lost their children, and 11 refused to continue further questions after the interview started. Of 36 children dead, 19, 11, and four children died at birth, before 28th day, and after 28th days after births, respectively. Two mothers did not remember when their children died. Therefore, the total number of participants in this study was 723 (Appendix 1).

### 2.3 Data collection

Data were collected using a structured, pretested, interviewer-administered questionnaire using surveyCTO, version 2.20 (Dobility Inc., Massachusetts). The baseline questionnaire was designed to collect data on socio-demographic characteristics, and other potential confounders such as enrollment in health insurance, decision-makers for health service utilizations. The follow-up questionnaire was designed to collect data on delivery, antenatal care, and other health-seeking behaviors. The basic questions were derived from the standard questionnaire for Demographic and Health Survey. The questionnaires for both baseline and follow-up surveys were pre-tested and revised accordingly.

### 2.4 Outcome and variables of interests

This study set delivery place as the primary outcome. The options for delivery place were composed of three, i.e., governmental health facility, private health facility, and other locations (e.g., traditional birth attendant’s clinic or residence, pregnant woman’s home, or church). Facility-based delivery is defined as a delivery taking place either at a governmental or private health facility where formally licensed health professionals (SBAs) such as doctors, nurses, and midwives are readily available, in this study.

To address the multifaced dimensions of the barriers to facility-based delivery, this study employed three proxy variables. First, wealth index was created by undertaking principal component analysis of the household's ownership of assets (i.e. electricity connection, radio, TV, mobile phone, refrigerator, generator, electric fan, air conditioner, personal computer, bicycle, motorcycle and vehicle), type of materials of housing structure (i.e. floor, roof, and external wall), type of source of drinking water, and type of sanitation facility. We used the first component as a wealth index in this study. Second, the respondent’s preferred language for interview was used to assess their linguistic barriers. Third, the locations of respondent’s residential area were used to evaluate their physical barrier. It is essential to ensure homogeneity among the households in a residential area, when addressing physical barriers. Thus, locations of households' residences were grouped not into EAs or government’s administrative boundaries, but into six clusters that were constructed by the hierarchal clustering method. The number of clusters was determined as six, based on mean misclassification error.

Fourteen potential confounders (Table 1) were controlled for multivariate analysis. Those potential confounders were composed of: (i) mothers’ age; (ii) fertility; (iii) ethnic group; (iv) education attainment; (v) English reading capacity; (vi) ownership of means of transportation (motorcycle/car/track); (vii) willingness to use facility-based health services; (viii) enrollment in health insurance; (ix) emergency preparedness; (x) decision-makers for health service utilizations; (xi) number of antenatal care visits; and (xii) locations of antenatal care services (governmental, private and others).

**Table 1:** Descriptive statistics of the participants before multiple imputation in Lagos Mainland LGA, Lagos State, Nigeria.

Total sample	723
**Delivery places (%)** (N = 554)	
Others (TBAs, homes, etc)	165 (29.8)
Governmental health facilities	232 (41.9)
Private health facilities	157 (28.3)

**Variables of Interest**	
Wealth score (mean (sd))	−0.00 (1.00)
Preferred language for interview (%)	
Yoruba	336 (46.5)
English	259 (35.8)
Egun	86 (11.9)
Igbo and Hausa	42 (5.8)
Location of households (%)	
Yaba/Onike/Alagomeji	100 (14.0)
Ebute-meta/Abule-Nla/Olaleye-Abule-Nla	159 (22.3)
Otto/Oyingbo	205 (28.8)
Makoko	131 (18.4)
Iwaya/Oko-Agbon	85 (11.9)
Abule-Ijesha	32 (4.5)

**Confounding variables**	
Age (mean (sd))	28.29 (5.68)
Fertility (mean (sd)) (N = 513)	2.17 (1.28)
Ethnic (%)	
Yoruba	388 (53.7)
Igbo	165 (22.8)
Egun	105 (14.5)
Others	65 (9.0)
Education (%)	
Never	107 (14.8)
Pre-primary	22 (3.0)
Primary	196 (27.1)
Secondary	300 (41.5)
Higher	98 (13.6)
English reading capacity (%)	
Unable	231 (32.0)
Partially able	140 (19.4)
Able	352 (48.7)
Means of transportation (%)	120 (16.6)
Willingness to use HFs (%)	667 (92.3)
Health insurance (%)	31 (4.3)
Preparation for emergency (%)	271 (37.5)
Decision maker for health (%)	
Myself	179 (24.8)
My husband/partner	400 (55.3)
Joint	78 (10.8)
Other	66 (9.1)
Number of ANC visit (mean (sd)) (N = 456)	9.30 (5.98)
ANC at private health facilities (%) (N = 575)	138 (19.1)
ANC at governmental health facilities (%) (N = 575)	291 (50.6)
ANC at other places (e.g., TBAs) (%) (N = 575)	93 (16.2)

### 2.5 Data analysis

Before performing statistical analyses, multiple imputations (MI) was performed using the R program, Amelia II [24], to address missing data at random. The variables applied to MI included the primary outcome variable, variables of interest, potential confounders, and the auxiliary variables. As a result, a total of 38 variables were employed in MI. The auxiliary variables for MI included occupation, hand washing practice, type of planned delivery places, partner’s presence, religion, number of years living in the current residential community, number of household members, number of children under five years of age, time spent walking from household’s residential area to the nearest health facility, perception towards distance as a barrier to health service utilization, herbal medicine usage, financial preparedness for delivery, preparation of delivery kits and transportation for delivery, and ownership of maternal health booklet or other home-based record. We created 20 full datasets using these variables for subsequent statistical analyses.

**Fig. 1:**
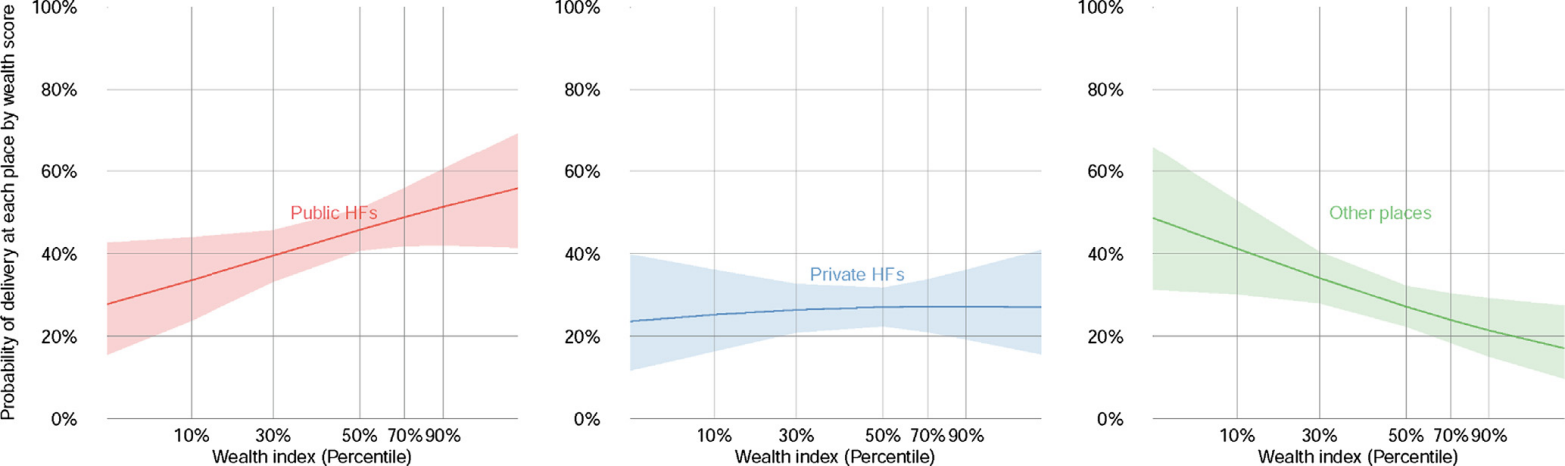
Probability of delivery places by wealth index. Based on the second model, we estimated the probabilities of delivery places by wealth index with holding the means of other covariates. The 95% CI of each probability was also described in this figure. 10th, 30th, 50th, 70th and 90th percentile indicate the middle level of the five groups respectively; poorest, poor, middle, rich and richest.

**Fig. 2:**
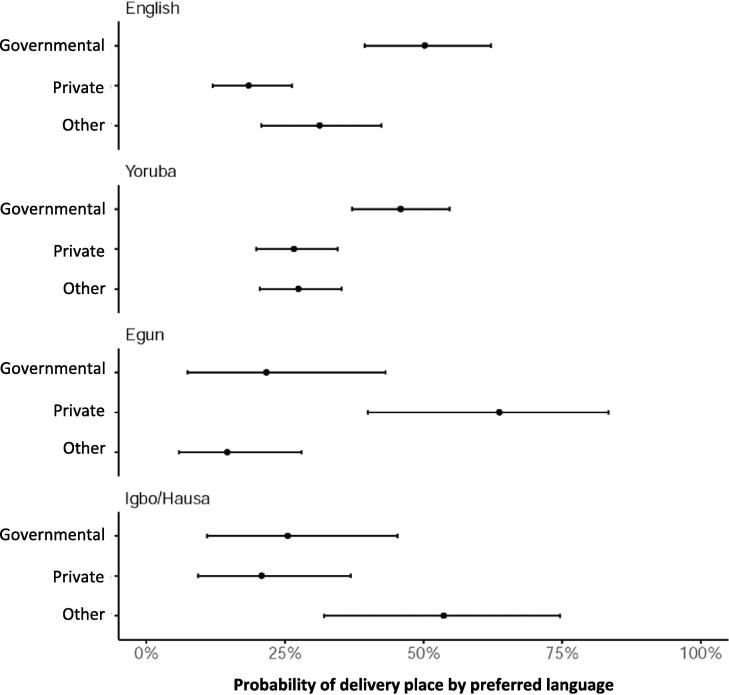
The estimated probabilities of delivery places by the fluent languages. Based on the second model, we estimated the probabilities of each delivery place by the first languages with holding the means of other covariates. The 95% CIs of each probability was also described in this figure as a horizontal bar.

The outcome variable (i.e., delivery place) is a categorical variable. Assuming no specific confounder for each delivery place, independence of irrelevant alternatives and invariant proportion of substitution, a multinomial logit analysis was selected. Fourteen confounders were included in the first model. Since the location of ANC produced a significant association exclusively with delivery places in the first model, ANC place was assumed to be an intermediate outcome between the outcome variable and the three proxy indicators of the socioeconomic barriers. Therefore, the second analysis without ANC related variables was conducted to remove an intermediate outcome. The estimated probabilities of respective delivery places were calculated by wealth index and preferred languages based on the second model entering the means of the other covariates. Fig. 1, Fig. 2 in the result section show the probabilities by wealth index and preferred languages.

We performed a sensitivity analysis without applying wealth index to assess any changes in associations between the outcome and other two proxy variables, preferred languages and residential area. Moreover, we conducted another model by replacing the residential area clusters for the government’s administrative boundaries. All the statistical analyses were performed by R Statistical Software (Foundation for Statistical Computing, Vienna, Austria)

## 3 Results

Table 1 shows the descriptive analyses of the outcome variable, the three factors of financial, linguistic and physical barriers, and 14 confounders. While 41.9% of the participants delivered at governmental health facilities, 28.3% and 29.8% delivered either at private health facilities or other places respectively. A great majority of them (82.3%) selected either English or Yoruba as languages they preferred for interview. Yoruba ethnic group accounted for 53.7% of the participants, being followed by Igbo (22.8%), Egun (14.5%) and other languages (9.0%). While most of Egun women (75.2%) preferred Egun as a language for interviews, 85.5% of Igbo women selected English. Most pregnant women (92.3%) were willing to utilize any types of health facilities. Only 4.3% of them were enrolled in a health insurance program. Note that 50.6%, 19.1% and 16.2% of the participants made at least one ANC visit either to governmental, private health facilities and other places, respectively.

Based on the first model including all fourteen confounders as well as ANC related factors, ANC places were significantly related to the delivery places (shown in Table 2). Those who visited governmental health facilities for ANC had significantly higher odds for delivery at a governmental health facility than those who didn’t (Adj. OR: 10.291, 95% CI: 4.329–24.465, p-value <0.001). Similarly, pregnant women utilizing private health facilities for ANC services were significantly more likely to deliver at a private health facilities (Adj. OR: 12.145, 95% CI: 5.095–28.948, p-value <0.001).

**Table 2:** Results of the multinomial logit regression analyses of delivery places with and without antenatal care-related variables in Lagos Mainland LGA, Lagos State, Nigeria.

	Others vs. Governmental health facilities		Others vs. Private health facilities	
Variables	Adj. OR.	95% CI	Adj. OR.	95% CI
**First model with ANC-relatedvariables**^1^				
**Number of ANC visits**	1.039	0.977–1.105	1.000	0.923–1.083
**ANC places**				
At private HFs	0.643	0.203–2.033	12.145***	5.095–28.948
At governmental HFs	10.291***	4.329–24.465	1.077	0.442–2.621
At other places	0.255**	0.098–0.662	0.328*	0.123–0.873

**Second model without ANC-relatedvariables**^2^				
**Wealth score**	1.522*	1.095–2.115	1.331	0.913–1.942
**Preferred language for interviews**				
Yoruba	Ref.	–	Ref.	–
English	0.964	0.437–2.127	0.604	0.264–1.379
Egun	0.855	0.247–2.959	4.772*	1.508–15.103
Igbo or Hausa	0.273*	0.079–0.94	0.382	0.118–1.234
**Location of households**				
Cluster 1	Ref.	–	Ref.	–
Cluster 2	0.848	0.474–1.515	1.092	0.578–2.061
Cluster 3	1.284	0.665–2.479	1.183	0.578–2.422
Cluster 4	0.675	0.267–1.706	0.63	0.222–1.789
Cluster 5	0.993	0.442–2.231	0.644	0.238–1.743
Cluster 6	2.156	0.684–6.789	2.485	0.746–8.274

Coef.: Coefficients, CI: Confidence interval

* < 0.05.

** <0.01.

*** <0.001.

1 In addition to ANC related variables, controlled by the other confounders as well as the three proxy indicators of the socio-economic barriers listed in the methods section.

2 Controlled by the other confounders listed in the methods section (mothers’ age; fertility; ethnic group; education attainment; English reading capacity; ownership of means of transportation; willingness to use facility-based health services; enrollment in health insurance; emergency preparedness; decision-makers for health service utilization).

Table 2 also shows the results of the second model that removed ANC related variables from the first model. Household wealth was positively associated with delivering at governmental health facilities (Adj. OR.: 1.522, 95% CI: 1.095–2.115, p-value <0.05). Women from wealthier households were more likely to deliver at governmental health facilities. Egun-speaking women were more likely to deliver at private health facilities than Yoruba-speaking women (Adj. OR.: 4.772, 95% CI: 1.508–15.103, p-value <0.05), while Igbo-speaking or Hausa-speaking women were more likely to utilize other delivery places than governmental health facilities (Adj. OR: 0.273, 95% CI: 0.079–0.94, p-value <0.05). There was no significant association between the outcome and the locations of households.

Fig. 1 presents the estimated probability of respective delivery places by wealth index based on the second model, and assuming that the means of other covariates. At the 10th percentile, the estimated probability of deliveries at other places was 41.2% (95% CI: 30.2–52.8%). The higher wealth index a household has, the greater probability of deliveries at governmental health facilities their women had from 33.5% (95% CI: 23.8–44.0%) among 10th percentile households to 51.4% (95% CI: 42.1–60.5% among 90th percentile households). The probabilities of deliveries at private health facilties were likely to remain the same across the wealth index (50th percentile: 27.1%, 95% CI: 22.5–33.7%).

The estimated probabilities of deliveries at respective places by the preferred languages are presented in Fig. 2. Governmental health facilities were the commonest option as delivery places among English-speaking (50.3%, 95% CI: 39.4–62.1%) and Yoruba-speaking women (45.9%, 95% CI: 37.2–54.7%). The probability of deliveries at private health facilities was the highest among Egun-speaking women (63.7%, 95% CI: 40.0–83.4%), while the probability of deliveries at other places was the highest among Igbo-speaking or Hausa-speaking women (53.7%, 95% CI: 32.1–74.7%).

Exclusion of wealth index from the second model did not influence the associations between the outcome variable and other two proxy factors such as languages and residential areas. In addition, the model that replaced the area clusters by the governmental administrative boundaries did not produce a different result from the second model.

## 4 Discussion

It was found that the proportions of deliveries at governmental and private health facilities were 41.9% and 28.3% respectively. Although the previous Demographic Health Survey 2013 reported higher utilization of private facilities for deliveries in Lagos State, Nigeria (56.1%) [3], an earlier study conducted in an LGA of Lagos State close to our study sites reported the proportions similar to our study (governmental health facilities: 40.2%, private health facilities: 19.7%) [5]. The government health facilities remain the commonest place for childbirths in the urban poor communities of Lagos.

This study found that ANC related variables were the significant predictors of delivery places. Earlier studies also reported that ANC visit to health facilities was one of the significant determinants of facility-based deliveries [25], [26], [27]. Women having made ANC visits to health facilities would have more opportunities to obtain knowledge on the importance of SBA attendance and delivery cost at health facilities. ANC at health facilities further provides pregnant women with a precious opportunity to build a trust relationship with facility-based health workers. ANC visits to traditional birth attendants (TBAs) similarly encourage pregnant women to deliver at their clinics. In this study, the commonest reason for delivering at TBA clinics or other places despite having initially planned to deliver at a health facility was continuous ANC visits to TBAs clinics. In the study sites, some TBAs provide more frequent ANC services (e.g., weekly-based services), accept more patient-friendly and flexible payment modes (e.g., installment payment and in-kind payment), and make themselves more readily available for pregnant women within their community whenever services are needed.

Based on the second model, the wealth index was significantly associated with deliveries at governmental health facilities. The gap in estimated probabilities of delivering at governmental health facilities between the lowest decile (the poorest) and the highest decile (the richest) was 17.9%. This result suggested that there remains the financial barrier to utilizations of governmental health facilities for delivery purpose among the urban poor in urban poor communities. The financial barrier prevents pregnant women from using safe delivery care at governmental health facilities.

Two thousand Nigerian Naira (NGN), equivalent to around five United States Dollar (USD), should be paid for a package of delivery care services at primary governmental health facilities according to the standard price list of health services defined by Lagos Primary Health Care Board. Yet, an additional fee (approx. NGN 3000) is charged for laboratory testing and ultrasound during ANC. Secondary and higher governmental hospitals commonly charge much higher than primary governmental health facilities such as NGN 20,000 or more for delivery care services. Enrollment in health insurance can serve as one of the financially protective interventions enabling financial barrier to be minimized. According to the Lagos State health insurance scheme having commenced in 2019, the total charge for comphensive delivery care (incl. Ceserian section, if needed) would range between NGN 8000 and NGN 40,000 depending on income level. It is worth assessing the impact of the newly launched health insurance on reduction in financial burden in delivery care [28]. This study also found that wealth level was not associated with private health facility utilizations for delivery care. This finding may be because this study did lump up all types of private health facilities into one group without categorizing them into several sub-groups, for instance, by a number of beds, types of health services readily available, quality of care, and prices of maternal care services. Further studies are needed to compare the financial costs and payment modes for delivery care between governmental health facilities, private health facilities, and traditional birth attendants.

The second variable of interest was the linguistic barrier for delivery care. Having controlled by ethnic groups, those having English reading capacity, higher education attainments, Hausa and Igbo language speakers who preferred to use them for interviews and conversations were significantly highly likely to deliver at other places other than health facilities. The probability of deliveries at private health facilities among women having selected Egun as preferred language was significantly higher than that among women having selected Yoruba. Exclusion of wealth index from the model did not influence the associations between delivery places and language types. Since the majority of health workers working at governmental health facilities were Yoruba, they commonly can speak both English and Yoruba. Thus, the linguistic barrier between Yoruba women and Yoruba-speaking health workers should be limited. Miscommunications between health workers and prengnat women derived from linguistic barrier possibly causes mismisunderstanding of diagnosis results and inappropriate treatment, which can impact patients’ outcome [29], [30]. The results of our study imply a greater need for building an environment friendly to Igbo and Hausa women. Employment of interpreters, telephone interpretation services, and use of IT-based interpretation equipment might be a possible solution for linguistic barriers [31]. Most health facilities in the study sites are equipped with a smartphone. Thus, for instance, the use of Google Translation that covers Hausa and Igbo languages would serve as a potential solution for linguistic barriers.

There was no significant association between delivery places and residential areas. While earlier studies found that distance to health facilities was one of the determinants of delivery care utilization [10], [32], this study did not find the similar result. However, the results of bivariate analysis between residential areas and delivery place indicated that those living in the city center were more likely to deliver at governmental health facilities than those living in waterfront urban slum areas. Identification of no association between delivery places and residential areas might have been attributable to the sample size not large enough to detect a significant association.

### 4.1 Strengths and limitations of the study

The major strength of the study is its study design, i.e., prospective cohort study whose sample size is representative of pregnant women living in urban slum areas of Lagos Mainland LGA. Possible confounders and intermediate outcome were controlled to assess the associations between the primary outcome and the variables of interest. Sensitivity analyses contributed to consolidating our argument.

One of the limitations of the study is that there are some missing data. Particularly, we had missing data on the primary outcome and ANC-related variables. To minimize this limitation, we assumed missing at random and performed multiple imputations. Another limitation is that this study was unable to collect data and information on the quality of maternal care services provided at respective health facilities and TBA clinics, which often could be a determinant of opting a delivery place.

Despite these limitations, we believe that the results of this study will be contributing enough to inform the policy decision on strategizing the way of addressing multifaced barriers to facility-based deliveries among pregnant women in urban poor communities of Lagos.

## 5 Conclusion

This study identified the financial and linguistic barriers to utilizations of health facilities for delivery care among women living in urban poor communies, of Lagos, Nigeria. Our results encourage policymakers and government officers to address these factors to provide skilled delivery care to a broader populations.

## Ethical approval

Ethical approval was obtained from the Health Research and Ethics Committee at Lagos State University Teaching hospital (Ref: LREC/06/10/764). Appropriate community entry was done through the community leaders. Participation in the study was informed of the purpose of this study and requested to participate in this study on a voluntary basis. Their informed consent was obtained before the interview.

## CRediT authorship contribution statement

**Yoshito Kawakatsu:** Conceptualization, Formal analysis, Methodology, Visualization, Writing - original draft. **Hirotsugu Aiga:** Methodology, Investigation, Project administration, Supervision, Writing - review & editing. **Osy Ubani:** Project administration, Validation. **Adefunke Oyeniyi Adesina:** Project administration, Validation. **Sumihisa Honda:** Methodology, Writing - review & editing. **Junko Otaki:** Project administration. **Nobuhiro Kadoi:** Investigation, Project administration, Supervision, Validation, Writing - review & editing.

## Declaration of Competing Interest

The authors declare that they have no known competing financial interests or personal relationships that could have appeared to influence the work reported in this paper.
