# Supplementary material for: Socioeconomic barriers to facility-based delivery in urban poor communities of Lagos: Wealth, linguistic capacity, and residential area
Source: Health Policy Open. 2020 Nov 11;1:100019. doi: 10.1016/j.hpopen.2020.100019 (PMC10297788; doi:10.1016/j.hpopen.2020.100019)
Supplement: Supplementary data 1 [file mmc1.docx]

Exclude;

- Refused (n=54)

- Moved (n=81)

- Unreachable (n=48)

- Deceased (n=1)

- Delivered before the interview (n=203)

**Appendix 1. Flow diagram of the sampling procedure**

Mothers with alive children

(n=698)

Exclude;

- Refused to continue (n=11)

Mothers who lost children

(n=36)

Final participants

(n=723)

Follow-up survey

Baseline survey

Enrolled mothers

(n=734)

Exclude;

- Rejected (n=78)

- Absent (n=15)

- Unreachable (n=33)

- Relocated (n=56)

- Deceased (n=2)

- Miscarriage (n=82)

4: not pregnant

Exclude;

- Not selected (n=1,112)

Randomly selected participants

(n=1,000)

Enrolled pregnant women

(n=2,112)

Identified pregnant women in study site

(n=2,499)
